# Confirmatory factor analysis of celebrity worship, digital literacy, and nostalgia: Dataset of Indonesians

**DOI:** 10.1016/j.dib.2020.106417

**Published:** 2020-10-17

**Authors:** Juneman Abraham, Moondore Madalina Ali, Esther Widhi Andangsari, Lisa Esti Puji Hartanti

**Affiliations:** a Psychology Department, Faculty of Humanities, Bina Nusantara University, Jakarta 11480, Indonesia; b Doctoral Student, Department of Communication, University of Vienna, 1010 Vienna, Austria; c School of Communication, Atma Jaya Catholic University of Indonesia, Jakarta 12930, Indonesia

**Keywords:** Celebrity, Psychology, Digital literacy, Factor analysis, Nostalgic emotion, Construct validity, Indonesia

## Abstract

This dataset is a measurement of Celebrity Worship (CWS), Digital Literacy (DL), and Nostalgia (NA). The participants were (1) For CWS, *N* = 3,223 people (181 males, 3042 females; *M*_age_ = 19.64 years old; *SD*_age_ = 3.13 years), (2) For DL, *N* = 482 people (225 males, 257 females; *M*_age_ = 25.16 years old; *SD*_age_ = 8.54 years), (3) For NA, *N* = 658 people (140 males, 518 females; *M*_age_ = 21.26 years old; *SD*_age_ = 1.93 years). The data was obtained using a survey via *Google Forms* in June 2018 and April–July 2020 in Indonesia. The analysis techniques were confirmatory factor analyses/CFA using LISREL and test of differences using JASP. The data could be used by the Indonesian Ministry of Communication and Informatics (KEMENKOMINFO), Indonesian Ministry of Education and Culture (KEMENDIKBUD), Indonesian Ministry of Youth and Sports (KEMENPORA), as well as social marketers to map the three constructs in Indonesian youth and to prevent the adverse effects and impacts of celebrity worship, digital illiteracy, and inappropriate mode of nostalgia.

## Specifications Table

**Table:** 

Subject	Psychometrics
Specific subject area	Quantitative Psychology
Type of data	Tables
How data were acquired	The survey method was conducted with the help of an online Indonesian-language questionnaire measuring three variables, i.e. (1) Celebrity Worship/CWS, (2) Digital Literacy/DL, and (3) Nostalgia/NA. The questionnaire in English and Indonesian language is provided as a supplementary file.
Data format	Raw Analyzed
Parameters for data collection	Informed consents were obtained from the participants, which explained that each questionnaire requires around 10 min to complete and that they have the right to refuse to participate in this study.
Description of data collection	The data was collected via *Google Forms* in June 2018 and April–June 2020.
Data source location	Primary location: Provinces inside Java Island, Indonesia. Secondary location: Provinces outside Java Island, Indonesia.
Data accessibility	Data is with the article.

## Value of the Data

• This dataset contributes to celebrity worship mapping in Indonesia, so that certain government bodies, non-governmental organizations (NGOs), and companies that have CSR (corporate social responsibility) funds for the development of Indonesian youth can use it for educational campaigns (e.g. notices or information sheets), to prevent detrimental outcomes from extreme celebrity worship, such as physical, psychological, economic, and social losses, including losses of lives through suicide.
• The presented dataset can be used as the guiding material to develop educational modules, training, algorithms, public service announcements, social movements, and other intervention programs on digital literacy for society as well as the government's leading sector of information and communication, i.e. the Indonesian Ministry of Communication and Informatics (KEMENKOMINFO). This dataset is socially significant in a period of worldwide uncontrolled information. By considering this dataset carefully, Indonesian youth can be empowered to find, evaluate, and responsibly use reliable/credible information, and to also practice basic of internet safety.
• Open data on nostalgia can become an important socioemotional capital for Indonesian people and has the potential, for further insights and development of experiments, to be correlated with other psychological variables that are harmful in individual and social life by positioning nostalgia as a buffer. For laypeople, the dataset on nostalgia can function as a reflection to not be trapped in reminiscing the past, and to use it as a positive coping mechanism in managing actual adversity.

## 1 Data Description

Confirmatory factor analyses (CFA) were done on three variables, i.e. Celebrity Worship (CWS), Digital Literacy (DL), and Nostalgia (NA). A few studies indicated that CWS is negatively correlated with DL [1], and NA is positively correlated with CWS [2]. However, this study does not aim to test the relationship between the variables, but rather to provide confirmatory factor analysis for each variable.

**Table 1:** Goodness of fit statistics.

Variable	Chi-square	*RMSEA*	90% CI for *RMSEA*(Lower; Upper)	*SRMR*	*CFI*	*AGFI*
Celebrity Worship	*χ*^2^(289, *N* = 3223) = 5176.04, *p* = 0.00	0.07	0.071; 0.074	0.07	0.97	0.87
Digital Literacy	*χ*^2^(721, *N* = 482) = 3223.27, *p* = 0.00	0.07	0.069; 0.075	0.01	0.93	0.76
Nostalgia	*χ*^2^(94, *N* = 658) =243.52, *p* = 0.00	0.05	0.042; 0.057	0.03	0.99	0.94

*Note*. Good fit criteria: *RMSEA* 〈 0.08; *SRMR* < 0.08; *CFI* 〉 0.90; *AGFI* > 0.90 [6]; any three of these criteria were met.

**Table 2:** Correlation Matrix between dimensions of Celebrity Worship (CWS).

Dimension	ES	IP	BP
**ES**	1		
**IP**	0.80	1	
***SE***	0.01		
***t***	93.89		
**BP**	0.40	0.76	1
***SE***	0.02	0.01	
***t***	21.38	62.03	

*Note. t* > |1.96| = *p* < 0.05 (CFA Output from LISREL 8.80). ES = Entertainment-social; IP = Intense-personal; BP = Borderline-pathological.

**Table 3:** Correlation Matrix between dimensions of Digital Literacy (DL).

Dimension	SF	PC	VI	PU	CD	SS
**SF**	1					
**PC**	0.92	1				
***SE***	0.07					
***t***	13.52					
**VI**	1.04	1.08	1			
***SE***	0.13	0.10				
***t***	8.12	11.10				
**PU**	1.07	0.96	1.13	1		
***SE***	0.07	0.03	0.10			
***t***	15.80	33.24	11.39			
**CD**	1.06	0.94	1.02	1.02	1	
***SE***	0.07	0.03	0.10	0.03		
***t***	15.27	29.35	10.41	35.56		
**SS**	1.07	0.97	0.94	0.98	0.98	1
***SE***	0.06	0.02	0.09	0.02	0.03	
***t***	16.77	41.48	10.47	43.85	38.38	

*Note. t* > |1.96| = *p* < 0.05 (CFA Output from LISREL 8.80). SF = To effectively search and find the necessary information; PC = To protect oneself from malicious and redundant content; VI = To verify and critically evaluate information using alternative sources of information; PU = To adequately perceive information and effectively use it; CD = To effectively and correctly disseminate information; SS= Specific skills, ability to use new media, use internet-services and technical devices.

Factors of Celebrity Worship (CWS) are Entertainment-social (ES), Intense-personal (IP), and Borderline-pathological (BP) [3]. Factors of Digital Literacy (DL) are: to effectively search and find the necessary information (SF); to protect oneself from malicious and redundant content (PC); to verify and critically evaluate information using alternative sources of information (VI); to adequately perceive information and effectively use it (PU); to effectively and correctly disseminate information (CD); and specific skills, ability to use new media, use internet-services and technical devices (SS) [4]. Factors of Nostalgia (NA) are Positive Affect (PA), Self-regard (SR), Social Connectedness (SC), and (4) Meaning in life (ML) [5].

**Table 4:** Correlation Matrix between dimensions of Nostalgia (NA).

Dimension	PA	SR	SC	ML
**PA**	1			
**SR**	0.83	1		
***SE***	0.02			
***t***	54.63			
**SC**	0.74	0.69	1	
***SE***	0.02	0.02		
***t***	34.56	28.44		
**ML**	0.68	0.73	0.71	1
***SE***	0.02	0.02	0.02	
***t***	28.00	34.44	30.73	

*Note. t* > |1.96| = *p* < 0.05 (CFA Output from LISREL 8.80). PA = Positive affect; SR = Self-regard; SC = Social connectedness; ML = Meaning in life.

**Table 5:** Covariance Matrix of Celebrity Worship (CWS).

Item	ES1	ES2	ES3	ES4	ES5	ES6	ES7	IP1	IP2	IP3
**ES1**	0.87									
**ES2**	0.55	0.89								
**ES3**	0.68	0.58	0.84							
**ES4**	0.56	0.64	0.60	0.83						
**ES5**	0.52	0.64	0.55	0.66	0.97					
**ES6**	0.58	0.59	0.59	0.61	0.64	0.94				
**ES7**	0.56	0.58	0.59	0.57	0.61	0.64	1.04			
**IP1**	0.29	0.35	0.30	0.32	0.36	0.35	0.34	1.05		
**IP2**	0.51	0.57	0.55	0.60	0.55	0.52	0.53	0.39	1.18	
**IP3**	0.32	0.40	0.35	0.39	0.39	0.39	0.38	0.43	0.47	1.16

*Note*. ES = Entertainment-social; IP = Intense-personal.

**Table 6:** Covariance Matrix of Digital Literacy (DL).

Item	SF1	SF2	SF3	PC1	PC2	PC3	PC4	PC5	PC6	PC7
**SF1**	0.06									
**SF2**	0.01	0.11								
**SF3**	0.01	0.02	0.08							
**PC1**	0.01	0.01	0.02	0.07						
**PC2**	0.02	0.01	0.01	0.02	0.23					
**PC3**	0.01	0.02	0.04	0.01	0.08	0.22				
**PC4**	0.02	0.03	0.03	0.01	0.02	0.03	0.12			
**PC5**	0.00	0.03	0.02	0.00	0.03	0.04	0.04	0.19		
**PC6**	0.02	0.03	0.02	0.01	0.01	0.02	0.03	0.04	0.08	
**PC7**	0.01	0.03	0.03	0.02	0.03	0.06	0.03	0.06	0.03	0.17

*Note*. SF = To effectively search and find the necessary information; PC = To protect oneself from malicious and redundant content.

**Table 7:** Covariance Matrix of Nostalgia (NA).

Item	PA1	PA2	PA3	PA4	SR1	SR2	SR3	SR4	SC1	SC2
**PA1**	1.38									
**PA2**	1.19	1.65								
**PA3**	1.12	1.41	1.74							
**PA4**	1.06	1.38	1.37	1.89						
**SR1**	0.75	0.97	1.07	1.07	1.56					
**SR2**	0.85	1.06	1.14	1.10	1.20	1.54				
**SR3**	0.82	1.02	1.08	1.08	1.10	1.16	1.47			
**SR4**	0.82	1.07	1.12	1.11	1.17	1.26	1.21	1.66		
**SC1**	0.76	0.98	0.89	0.98	0.76	0.79	0.85	0.92	1.67	
**SC2**	0.84	1.05	0.95	1.02	0.79	0.82	0.81	0.92	1.45	1.83

*Note*. PA = Positive affect; SR = Self-regard; SC = Social connectedness.

The Goodness of Fit Statistics of each variable are presented in Table 1. The CFA illustrations are included in the Supplementary Materials (standardized and *t* values presented).

The correlation matrices between dimensions are shown in Table 2 (Celebrity Worship), Table 3 (Digital Literacy), and Table 4 (Nostalgia).

The covariance matrices of the latent variables are shown in the Supplementary Materials, and examples of the matrices can be found in Table 5 (Celebrity Worship), Table 6 (Digital Literacy), and Table 7 (Nostalgia). The covariance matrix allows data validation and extension by other data miners and interpreters, and gives a chance for other possible elucidations to be examined subsequently [7].

The correlations between age, all predictors, and criterion variables are shown in Table 8. The test of differences based on domicile is presented in Table 9, and descriptive statistics of the variables based on domicile is presented in Table 10. It is shown that age correlates positively with Celebrity Worship (as a total score) and all its dimensions, but correlates negatively (although low) with Digital Literacy (as a total score). In terms of CWS and two of its dimensions (ES/Entertainment-social, IP/Intense-personal), it is shown that samples outside Java scored higher than samples inside Java. Samples inside Java also scored higher than samples outside Java in terms of PC and PU but not Digital Literacy (as a total score).

**Table 8:** Pearson correlations (*r*) between age and the variables.

Variable	Standardized Cronbach's *α*	Average interitem correlation	*M*	*SD*	Correlation (*r*) with Age	*p*	95% CI for *r*(Lower; Upper)	*n*_item_	*N*
CWS_Total	0.93	0.35	2.81	0.82	0.27	<0.001	0.24; 0.30	26	3223
CWS_ES	0.92	0.66	3.75	0.21	0.32	<0.001	0.29; 0.35	7	3223
CWS_IP	0.89	0.39	2.76	0.53	0.25	<0.001	0.22; 0.28	13	3223
CWS_BP	0.77	0.35	1.82	0.46	0.07	<0.001	0.04; 0.11	6	3223
DL_Total	0.92	0.22	0.83	0.10	−0.17	<0.001	−0.25; −0.08	40	482
DL_SF	0.39*	0.18	N/R	N/R	N/R	N/R	N/R	3	482
DL_PC	0.70	0.21	N/R	N/R	N/R	N/R	N/R	9	482
DL_VI	0.33*	0.14	N/R	N/R	N/R	N/R	N/R	3	482
DL_PU	0.71	0.21	N/R	N/R	N/R	N/R	N/R	9	482
DL_CD	0.66	0.28	N/R	N/R	N/R	N/R	N/R	5	482
DL_SS	0.80	0.27	N/R	N/R	N/R	N/R	N/R	11	482
NA_Total	0.96	0.60	4.90	0.21	−0.02	0.62	−0.10; 0.06	16	658
NA_PA	0.93	0.76	4.88	0.25	−0.01	0.80	−0.09; 0.07	4	658
NA_SR	0.93	0.76	4.84	0.06	−0.01	0.78	−0.09; 0.07	4	658
NA_SC	0.91	0.72	4.74	0.18	−0.01	0.73	−0.09; 0.06	4	658
NA_ML	0.94	0.81	5.15	0.05	−0.03	0.39	−0.11; 0.04	4	658

*Note. N/R* = not relevant because of nominal variable (Output from JASP for Windows ver. 0.11.1). * Cronbach's *α* < 0.70 (low internal consistency). CWS = Celebrity Worship; DL = Digital Literacy; NA = Nostalgia; ES = Entertainment-social; IP = Intense-personal; BP = Borderline-pathological; SF = To effectively search and find the necessary information; PC = To protect oneself from malicious and redundant content; VI = To verify and critically evaluate information using alternative sources of information; PU = To adequately perceive information and effectively use it; CD = To effectively and correctly disseminate information; SS= Specific skills, ability to use new media, use internet-services and technical devices; PA = Positive affect; SR = Self-regard; SC = Social connectedness; ML = Meaning in life.

**Table 9:** Independent sample *t*-tests based on domicile.

						95% CI for *MD*		
Variable	*t*	*df*	*p*	*MD*	*SE* Difference	Lower	Upper	Cohen's *d*
CWS_ES	−3.35	3221	<.001	−0.79	0.24	−1.26	−0.33	−0.14
CWS_IP	−3.52	3221	<.001	−1.38	0.39	−2.15	−0.61	−0.15
CWS_BP	−1.21	3221	0.23^a^	−0.19	0.16	−0.50	0.12	−0.05
CWS_Total	−3.48	3221	<.001	−2.36	0.68	−3.70	−1.03	−0.15
DL_SF	0.85	480	0.40	0.05	0.05	−0.06	0.15	0.08
DL_PC	−2.04	480	0.04	−0.36	0.18	−0.71	−0.01	−0.19
DL_VI	−0.43	480	0.67	−0.03	0.06	−0.15	0.10	−0.04
DL_PU	−2.02	480	0.04	−0.34	0.17	−0.67	−0.01	−0.18
DL_CD	−0.34	480	0.74	−0.03	0.10	−0.24	0.17	−0.03
DL_SS	0.29	480	0.77	0.05	0.18	−0.31	0.41	0.03
DL_Total	−1.06	480	0.29	−0.67	0.63	−1.89	0.56	−0.10

*Note. MD* = *Mean Difference. SE = Standard Error.*^a^ Levene's test is significant (*p* < 0.05), suggesting a violation of the equal variance assumption (Output from JASP for Windows ver. 0.11.1). CWS = Celebrity Worship; DL = Digital Literacy; NA = Nostalgia; ES = Entertainment-social; IP = Intense-personal; BP = Borderline-pathological; SF = To effectively search and find the necessary information; PC = To protect oneself from malicious and redundant content; VI = To verify and critically evaluate information using alternative sources of information; PU = To adequately perceive information and effectively use it; CD = To effectively and correctly disseminate information; SS= Specific skills, ability to use new media, use internet-services and technical devices; PA = Positive affect; SR = Self-regard; SC = Social connectedness; ML = Meaning in life.

**Table 10:** Descriptive statistics based on domicile.

Variable	Domicile	*N*	*M*	*SD*	*SE*
CWS_Total	Java Island	2502	72.47	15.85	0.32
	Outside Java Island	721	74.84	16.81	0.63
CWS_ES	Java Island	2502	26.06	5.58	0.11
	Outside Java Island	721	26.85	5.69	0.21
CWS_IP	Java Island	2502	35.57	9.17	0.18
	Outside Java Island	721	36.95	9.62	0.36
CWS_BP	Java Island	2502	10.85	3.66	0.07
	Outside Java Island	721	11.04	3.97	0.15
DL_Total	Java Island	453	33.57	6.46	0.30
	Outside Java Island	29	30.07	11.03	2.05
DL_SF	Java Island	453	2.74	0.55	0.03
	Outside Java Island	29	2.48	0.91	0.17
DL_PC	Java Island	453	7.14	1.90	0.09
	Outside Java Island	29	6.28	2.56	0.48
DL_VI	Java Island	453	2.49	0.66	0.03
	Outside Java Island	29	2.31	1.00	0.19
DL_PU	Java Island	453	7.25	1.78	0.08
	Outside Java Island	29	6.48	2.64	0.49
DL_CD	Java Island	453	4.31	1.08	0.05
	Outside Java Island	29	3.97	1.59	0.30
DL_SS	Java Island	453	9.64	1.90	0.09
	Outside Java Island	29	8.55	3.12	0.58

*Note.* CWS = Celebrity Worship; DL = Digital Literacy; NA = Nostalgia; ES = Entertainment-social; IP = Intense-personal; BP = Borderline-pathological; SF = To effectively search and find the necessary information; PC = To protect oneself from malicious and redundant content; VI = To verify and critically evaluate information using alternative sources of information; PU = To adequately perceive information and effectively use it; CD = To effectively and correctly disseminate information; SS= Specific skills, ability to use new media, use internet-services and technical devices; PA = Positive affect; SR = Self-regard; SC = Social connectedness; ML = Meaning in life.

## 2 Experimental Design, Materials and Methods

The data came from a psychometric design to assess the construct validity of Celebrity Worship, Digital Literacy, and Nostalgia. The survey methods and purposive sampling techniques were employed. Participant demographics are shown in Table 11, and the completed instruments are shown in Table 12. The data was analyzed with LISREL 8.80 in the form of Confirmatory Factor Analyses (CFA), correlation matrices, and covariance matrices, as well as JASP for Windows ver. 0.11.1. presented as descriptive statistics and tests of differences between domiciles. Confirmatory Factor Analysis is the appropriate method for construct validation by taking into account the relationship between latent factors and observed variables, so that it potentially reduces measurement errors and thus increases statistical accuracy [8].

**Table 11:** The participants.

Demography	Descriptive Statistics
Celebrity Worship (CWS)	*N* = 3223
Domicile	Jakarta & Banten = 897 (27.83%) Outside Jakarta & Banten (inside Java Island) = 1605 (49.80%) Outside Jakarta & Banten (outside Java Island) = 721 (22.37%)
Age	*M*_age_ = 19.64 years old; *SD*_age_ = 3.13 years
Sex	181 (5.62%) males, 3042 (94.38%) females*
Digital Literacy (DL)	*N* = 482
Domicile	Jakarta & Banten = 338 (70.13%) Outside Jakarta & Banten (inside Java Island) = 115 (23.85%) Outside Jakarta & Banten (outside Java Island) = 29 (6.02%)
Age	*M*_age_ = 25.16 years old; *SD*_age_ = 8.54 years
Sex	225 (46.68%) males, 257 (53.32%) females
Nostalgia (NA)	*N* = 658
Domicile	Jakarta & Banten = 285 (43.31%) Outside Jakarta & Banten (inside Java Island) = 373 (56.69%) Outside Jakarta & Banten (outside Java Island) = 0 (0.00%)
Age	*M*_age_ = 21.26 years old; *SD*_age_ = 1.93 years
Sex	140 (21.28%) males, 518 (78.72%) females

*Note*. * The imbalance between the number of sexes is due to the majority of participants who were *K-Pop Fans*[9] are females. Potential re-use of this dataset is limited, i.e. more applicable to females than males.

**Table 12:** The materials.

Instrument for Measuring the Variable	Scale's Dimensions and Items (see Supplementary Materials for the complete Questionnaire; items randomly ordered)	Response options and Scoring
Celebrity Worship/CWS Scale [3].	Dimensions: • Entertainment-social (Scale no. 1–7; code: ES1 – ES7): Attraction to celebrity as one's means of entertaining and for socializing with friends. • Intense-personal (Scale no. 8–20; code: IP1 – IP13): One's intense and compulsive feeling related to a celebrity. • Borderline-pathological (Scale no. 21–26; code: BP1 – BP6): Extreme attitude and behavior towards a celebrity as a form of maladaptive admiration. Filler item (Scale no. 27–34, not coded) Example of items: Keeping up with news about my favorite celebrity is an entertaining pass-time.	Response options: from “*Strongly Disagree”* (scored 1) to “*Strongly Agree*” (scored 5). Scoring: The higher the total CWS score, the higher the Celebrity Worship. Data were taken in June 2018 and April-June 2020.
Digital Literacy/DL Scale [4]	Dimensions: • To effectively search and find the necessary information (Scale no. 1–3; code: SF1 – SF3) • To protect oneself from malicious and redundant content (Scale no. 4–12; code: PC1 – PC9) • To verify and critically evaluate information using alternative sources of information (Scale no. 13–15; code: VI1 – VI3) • To adequately perceive information and effectively use it (Scale no. 16–24; code: PU1 – PU9) • To effectively and correctly disseminate information (Scale no. 25–29; code: CD1 – CD5) • Specific skills, ability to use new media, use internet-services and technical devices (Scale no. 30–40; code SS1 – SS11) Example of items: I see myself … know exactly whose interests the media (newspapers, magazines, TV, radio, Internet) represents - to evaluate information from the media.	Response options: Yes (scored 1) or No (scored 0) Scoring: The higher the total DL score, the higher the digital literacy. Data were taken in June-July 2020.
Nostalgia/NA Scale [5]	Dimensions: • Positive affect (Scale no. 1–4; code: PA1 – PA4): Nostalgia could reduce negative moods. • Self-regard (Scale no. 5–8; code: SR1 – SR4): Nostalgia could protect oneself from the threat of a defensive response, so that self-esteem increases. • Social connectedness (Scale no.9–12; code SC1 – SC4): Nostalgia could increase perceptions of social bonds, interpersonal skills and social support. • Meaning in life (Scale no. 13–16; code: ML1 – ML4): Nostalgia could improve the perception of life and relieve anxiety Example of items: Thinking about the most nostalgic event … puts me in a good mood	Response options: from “*Strongly Disagree”* (scored 1) to “*Strongly Agree*” (scored 6). Scoring: The higher the total NA score, the more positive the nostalgic emotion. Data were taken in April-June 2020.

## 3 Ethics Statement

Informed consents were obtained from the participants via the online questionnaires. This study was approved by the Bina Nusantara University International Research Scheme (in Indonesian: *Penelitian Internasional BINUS*/PIB) with the Research Contract Letter No. 026/VR.RTT/IV/2020. The ethical decree is stated in Article 1 Paragraph 3 of the Letter.

## CRediT Author Statement

Juneman Abraham: Conceptualization, Methodology, Validation, Formal analysis, Writing - Original

Draft, Writing - Review & Editing, Supervision

Moondore Madalina Ali: Conceptualization, Methodology, Validation, Formal analysis, Writing - Original Draft

Esther Widhi Andangsari: Conceptualization, Methodology, Validation, Formal analysis, Writing -

Original Draft

Lisa Esti Puji Hartanti: Conceptualization, Methodology, Validation, Formal analysis, Writing - Original Draft

## Declaration of Competing Interest

The authors declare that they have no known competing financial interests or personal relationships which have, or could be perceived to have, influenced the work reported in this article.

## Appendix Supplementary materials

Image, application 1

Image, application 2

## Footnotes

Supplementary material associated with this article can be found in the online version at doi:10.1016/j.dib.2020.106417.
