# Supplementary material for: Confirmatory factor analysis of celebrity worship, digital literacy, and nostalgia: Dataset of Indonesians
Source: Data Brief. 2020 Oct 17;33:106417. doi: 10.1016/j.dib.2020.106417 (PMC7586072; doi:10.1016/j.dib.2020.106417)
Supplement: Supplementary file 1 [file mmc1.docx]

**The Questionnaires of *Confirmatory Factor Analyses of Celebrity Worship, Digital Literacy, and Nostalgia: Dataset of Indonesians***

Declaration of Participation

By giving a checkmark (“ V “) in the column below, I declare that I agree to participate in this research.

There is no pressure on me to participate in this research. I realize that (1) I have the right to refuse if I don’t want to follow it, (2) Confidentiality of all my data and answers will be guaranteed for research purposes only, (3) The answers I give must be true to myself.

(*Dengan memberikan tanda cek* ‘V’ *pada tempat yang tersedia, saya menyatakan bahwa saya setuju untuk berpartisipasi dalam penelitian ini.*

*Tidak ada tekanan kepada saya untuk mengikuti penelitian ini. Saya menyadari bahwa (1) Saya berhak menolak jika tidak bersedia mengikutinya, (2) Semua data dan jawaban saya akan terjamin kerahasiaannya hanya untuk kepentingan penelitian, (3) Jawaban yang saya berikan harus benar-benar sesuai dengan diri saya*.)

(............................................)

Research Participant

Date :

Sex : Male | Female

Age :

Domicile :

INSTRUCTION:

The purpose of this survey is to identify your views about famous persons. The responses you give are confidential. There are no right or wrong answers, so please answer as openly and thoughtfully as you can.

*(Tujuan dari survei ini adalah untuk mengindentifikasi pandangan Anda tentang orang-orang terkenal. Tanggapan yang anda berikan bersifat rahasia. Tidak ada jawaban yang benar atau salah, silakan menjawab secara terbuka dan teliti.*)

For purposes of the survey we are defining the term “celebrity” as a famous living person (or one who died during your lifetime) that you greatly admire.

(*Untuk tujuan survei kami mendefinisikan istilah “selebriti” sebagai orang terkenal yang hidup (atau orang yang telah meninggal selama hidup anda) yang sangat anda kagumi.*)

Who is your favorite celebrity? ____________________ (Please choose one famous person, as defined above).

(*Siapa selebriti favoritemu? __________________ (Silakan pilih satu orang terkenal, sebagaimana di definisikan diatas)*)

Just in case your favorite celebrity is unknown to us, please select one or more of the following to describe why your favorite celebrity is famous:

Acting

Author

Artist

Medicine

Modeling

Music

News

Politics

Religion

Royalty

Radio or TV Talk Show

Science

Sports

Other (please describe)_________________

(*Apabila selebriti favorit anda tidak dikenal oleh kami, harap melingkari satu atau lebih dari yang berikut untuk menjelaskan mengapa selebriti favorite anda terkenal:*

*Akting*

*Penulis*

*Artis*

*Kedokteran*

*Model*

*Musik*

*Berita*

*Politik*

*Agama*

*Kebangsaan*

*Penyiar Radio atau TV*

*Ilmuwan*

*Olahragawan*

*Lainnya _________*)

| **No** | **Statement** | **Strongly disagree** | **Disagree** | **Uncertain or neutral** | **Agree** | **Strongly agree** |
| --- | --- | --- | --- | --- | --- | --- |
| 1 | I love to talk with others who admire my favorite celebrity  (*Saya senang berbicara dengan orang yang juga mengagumi selebriti idola yang sama dengan saya*) |  |  |  |  |  |
| 2 | Keeping up with news about my favorite celebrity is an entertaining pass-time  (*Mencari berita tentang selebriti idola saya adalah hobi yang menghibur*) |  |  |  |  |  |
| 3 | It is enjoyable just to be with others who like my favorite celebrity  (*Bersama dengan orang yang juga menyukai selebriti idola saya adalah hal yang menyenangkan*) |  |  |  |  |  |
| 4 | I enjoy watching, reading, or listening to my favorite celebrity because it means a good time  (*Saya menikmati waktu menonton, membaca, atau mendengarkan hal yang berkaitan dengan selebriti idola saya karena hal tersebut menyenangkan*) |  |  |  |  |  |
| 5 | Learning the life story of my favorite celebrity is a lot of fun  (*Mempelajari tentang kisah hidup selebriti idola saya adalah hal yang menyenangkan*) |  |  |  |  |  |
| 6 | I like watching and hearing about my favorite celebrity when I am in a large group of people  (*Saya senang mengamati dan mendengar orang-orang yang membicarakan selebriti idola saya*) |  |  |  |  |  |
| 7 | My friends and I like to discuss what my favorite celebrity has done  (*Saya dan teman-teman suka membahas apa yang telah dilakukan selebriti idola saya*) |  |  |  |  |  |
| 8 | If I were to meet my favorite celebrity in person, he/she would already somehow know that I am his/her biggest fan  (*Jika saya bertemu selebriti idola saya secara pribadi, ia akan tahu bahwa sayalah fans terbesarnya*) |  |  |  |  |  |
| 9 | One of the main reasons I maintain an interest in my favorite celebrity is that doing so gives me a temporary escape from life’s problems  (*Salah satu alasan saya memiliki ketertarikan terhadap selebriti idola adalah karena dapat membuat saya melupakan masalah untuk sementara*) |  |  |  |  |  |
| 10 | My favorite celebrity is practically perfect in every way  (*Selebriti idola saya sempurna dalam segala hal*) |  |  |  |  |  |
| 11 | I share with my favorite celebrity a special bond that cannot be described in words  (*Saya memiliki ikatan khusus dengan selebriti idola saya yang tidak dapat dijelaskan dengan kata-kata*) |  |  |  |  |  |
| 12 | To know my favorite celebrity is to love him/her  (*Bentuk cinta saya kepada selebriti idola adalah dengan mencari tahu tentang selebriti idola saya*) |  |  |  |  |  |
| 13 | When something bad happens to my favorite celebrity I feel like it happened to me  (*Ketika sesuatu yang buruk terjadi pada selebriti idola saya, saya merasa itu juga terjadi pada saya*) |  |  |  |  |  |
| 14 | When my favorite celebrity fails or loses at something I feel like a failure myself  (*Ketika selebriti idola saya mengalami kegagalan, saya juga merasa gagal*) |  |  |  |  |  |
| 15 | The successes of my favorite celebrity are my successes also  (*Kesuksesan selebriti idola saya adalah kesuksesan saya juga*) |  |  |  |  |  |
| 16 | I consider my favorite celebrity to be my soulmate  (*Saya menganggap selebriti idola saya adalah belahan jiwa saya*) |  |  |  |  |  |
| 17 | When my favorite celebrity dies (or died) I will feel (or I felt) like dying too  (*Ketika selebriti idola saya meninggal, saya akan merasa seolah mati juga*) |  |  |  |  |  |
| 18 | If someone gave me several thousand dollars to do with as I please, I would consider spending it on a personal possession (like a napkin or paper plate) once used by my favorite celebrity  (*Jika seseorang memberi saya beberapa jumlah uang untuk melakukan apa yang saya inginkan, saya akan mempertimbangkan untuk membeli barang yang pernah digunakan oleh selebriti idola saya*) |  |  |  |  |  |
| 19 | When something good happens to my favorite celebrity I feel like it happened to me  (*Ketika sesuatu yang baik terjadi pada selebriti idola saya, saya merasa itu juga terjadi pada saya*) |  |  |  |  |  |
| 20 | I am obsessed by details of my favorite celebrity’s life  (*Saya terobsesi dengan seluruh kehidupan selebriti idola saya*) |  |  |  |  |  |
| 21 | I would gladly die in order to save the life of my favorite celebrity  (*Saya rela berkorban bahkan sampai mati untuk menyelamatkan selebriti idola saya*) |  |  |  |  |  |
| 22 | If I were lucky enough to meet my favorite celebrity, and he/she asked me to do something illegal as a favor, I would probably do it  (*Jika saya cukup beruntung untuk bertemu selebriti idola saya dan dia meminta saya untuk melakukan sesuatu yang ilegal untuk menolongnya, saya mungkin akan melakukannya*) |  |  |  |  |  |
| 23 | If I walked through the door of my favorite celebrity's home without an invitation she or he would be happy to see me  (*Jika saya masuk ke rumah selebriti idola saya tanpa diundang, dia akan senang melihat saya*) |  |  |  |  |  |
| 24 | I have frequent thoughts about my favorite celebrity, even when I don’t want to  (*Saya sering terpikir selebriti idola saya, bahkan ketika saya sedang tidak ingin memikirkan selebriti idola saya*) |  |  |  |  |  |
| 25 | My favorite celebrity would immediately come to my rescue if I needed help  (*Jika saya membutuhkan pertolongan, selebriti idola saya akan datang untuk menyelamatkan saya*) |  |  |  |  |  |
| 26 | My favorite celebrity and I have our own code so we can communicate with each other secretly (such as over the TV or special words on the radio)  (*Saya dan selebriti idola saya mempunyai kode sendiri, sehingga kami dapat berkomunikasi satu sama lain secara diam-diam (seperti di TV atau kata-kata special di radio)*) |  |  |  |  |  |
| 27 | I often feel compelled to learn the personal habits of my favorite celebrity  (*Saya selalu terdorong untuk mempelajari kebiasaan pribadi dari selebriti idola saya*) |  |  |  |  |  |
| 28 | I have pictures and/or souvenirs of my favorite celebrity which I always keep in exactly the same place  (*Saya punya gambar / souvenir dari selebriti idola saya yang selalu disimpan di tempat yang sama*) |  |  |  |  |  |
| 29 | If my favorite celebrity was accused of committing a crime that accusation would have to be false  (*Jika selebriti idola saya dituduh melakukan kejahatan, menurut saya tuduhan tersebut tidak benar*) |  |  |  |  |  |
| 30 | If my favorite celebrity endorsed a legal but possibly unsafe drug designed to make someone feel good, I would try it  (*Jika selebriti idola saya membintangi iklan obat legal, tetapi obat tersebut memiliki efek samping yang membahayakan, saya akan mencobanya*) |  |  |  |  |  |
| 31 | News about my favorite celebrity is a pleasant break from a harsh world  (*Berita tentang selebriti idola saya adalah hiburan yang menyenangkan*) |  |  |  |  |  |
| 32 | If my favorite celebrity found me sitting in his/her car he or she would be upset  (*Jika selebriti idola saya menemukan saya duduk di dalam mobilnya, dia akan marah*) |  |  |  |  |  |
| 33 | It would be great if my favorite celebrity and I were locked in a room for a few days  (*Akan sangat menyenangkan jika selebriti idola saya dan saya terkunci di sebuah ruangan selama beberapa hari*) |  |  |  |  |  |
| 34 | If my favorite celebrity saw me in a restaurant he/she would ask me to sit down and talk  (*Jika selebriti idola saya melihat saya di sebuah restoran, dia akan meminta saya untuk duduk dan ngobrol dengannya*) |  |  |  |  |  |

INSTRUCTION:

Do you see yourself ...?

*(Apakah Anda memandang diri Anda ...?)*

| **No** | **Statement** | **Yes** | **No** |  |  |  |  |
| --- | --- | --- | --- | --- | --- | --- | --- |
| 1 | Are able to collect information from different sources in order to get a full picture of the topic of interest  (*Mampu mengumpulkan informasi dari berbagai sumber untuk mendapatkan gambaran lengkap tentang topik yang sedang diperhatikan*) |  |  |  |  |  |  |
| 2 | Can correctly formulate search queries in Google etc.  (*Dapat dengan benar merumuskan permintaan pencarian di Google, dan sebagainya*) |  |  |  |  |  |  |
| 3 | Are able to maximally use Internet search services (Google, etc.)  (*Mampu menggunakan layanan pencarian Internet (Google, dll.) secara maksimal*) |  |  |  |  |  |  |
| 4 | Know exactly how information received from the media can influence the thoughts and behavior of a person  (*Mengetahui persis bagaimana mekanisme informasi yang diterima dari media dapat memengaruhi pikiran dan perilaku seseorang*) |  |  |  |  |  |  |
| 5 | Familiar with the noon of “manipulation with information”  (*Akrab dengan gagasan “manipulasi dengan informasi”*) |  |  |  |  |  |  |
| 6 | Familiar with the concept of “computer viruses”  (*Akrab dengan konsep “virus komputer”*) |  |  |  |  |  |  |
| 7 | Are able to clean the computer of junk files  (*Mampu membersihkan komputer dari file sampah*) |  |  |  |  |  |  |
| 8 | Can check the computer for viruses  (*Dapat memeriksa komputer apakah ada virus*) |  |  |  |  |  |  |
| 9 | Can make backup copies of files stored on the computer  (*Dapat membuat salinan cadangan file yang disimpan di komputer*) |  |  |  |  |  |  |
| 10 | Are able to recognize the situation of blackmail of information in the Internet  (*Mampu mengenali situasi pemerasan informasi di Internet*) |  |  |  |  |  |  |
| 11 | Are able to determine the degree of confidentiality and security of the transfer of personal data using the services via the Internet  (*Mengetahui persis tingkat kerahasiaan dan keamanan transfer data pribadi dalam berbagai layanan di Internet*) |  |  |  |  |  |  |
| 12 | Can use the function of parental control on the computer and online services  (*Dapat menggunakan fungsi kontrol orang tua di komputer dan layanan online*) |  |  |  |  |  |  |
| 13 | Agree that newspapers, radio stations, television channels and Internet portals can be both private and public  (*Setuju bahwa surat kabar, stasiun radio, saluran televisi dan portal Internet dapat bersifat publik (mewakili kepentingan umum) maupun privat (mewakili kepentingan perusahaan/pribadi tertentu)*) |  |  |  |  |  |  |
| 14 | Can notice the differences between information and other messages - opinions, judgments, criticism  (*Mengetahui persis perbedaan antara informasi dan jenis pesan lain (opini, penilaian, kritik)*) |  |  |  |  |  |  |
| 15 | Are able to reveal the hidden informaon about additional payments for the use of the service  (*Mampu mengungkapkan informasi tersembunyi tentang pembayaran tambahan untuk penggunaan layanan tertentu*) |  |  |  |  |  |  |
| 16 | Familiar with the concept of “plagiarism”  (*Akrab dengan konsep “plagiarisme”*) |  |  |  |  |  |  |
| 17 | Familiar with the concept of “information war”  (*Akrab dengan konsep “perang informasi”*) |  |  |  |  |  |  |
| 18 | Akrab dengan konsep “kecanduan jaringan”  (*Familiar with the concept of “network addicon”*) |  |  |  |  |  |  |
| 19 | Familiar with the concept of “compromising evidence”  (*Akrab dengan konsep “bukti-bukti yang dilonggarkan kriteria keakuratannya”*) |  |  |  |  |  |  |
| 20 | Agree that the Internet primarily provides opportunies  for the transfer of professional and educational information  (*Setuju bahwa Internet terutama memberi peluang untuk transfer informasi profesional dan pendidikan*) |  |  |  |  |  |  |
| 21 | Are able to determine whether the information found in the Internet is trustworthy  (*Mampu menentukan apakah informasi yang ditemukan di Internet dapat dipercaya*) |  |  |  |  |  |  |
| 22 | Are able to recognize the indication that a certain program is suitable for children and adolescents  (*Mampu mengenali indikasi bahwa suatu program digital tertentu cocok untuk anak-anak dan remaja*) |  |  |  |  |  |  |
| 23 | Are able to determine what information contains advertising  (*Mampu menentukan informasi seperti apa yang mengandung iklan*) |  |  |  |  |  |  |
| 24 | Know exactly whose interests the media (newspapers, magazines, TV, radio, Internet) represents - to evaluate information from the media  (*Mengetahui persis minat/kepentingan siapa yang diwakili sebuah media (surat kabar, majalah, TV, radio, Internet) - untuk mengevaluasi informasi dari media tersebut*) |  |  |  |  |  |  |
| 25 | Agree that the usage of electronic means of communicaon (sites, social networks, etc.) always implies the collection of personal data  (*Mengetahui persis bagaimana penggunaan alat komunikasi elektronik (situs, jejaring sosial, dll.) mengandung pengumpulan data pribadi*) |  |  |  |  |  |  |
| 26 | Agree that anonymity on the Internet is often deceptive - each user can be identified  (*Mengetahui persis bagaimana anonimitas di Internet (bahwa orang tidak akan dapat dikenali kalau tidak memasang nama di Internet) sering menipu - bahwa masing-masing pengguna internet sebenarnya dapat dikenali/diidentifikasi*) |  |  |  |  |  |  |
| 27 | Agree that posting some of the messages in the Internet can  negavely affect one’s career and personal life  (*Mengetahui bagaimana memposting pesan tertentu di Internet dapat secara negatif memengaruhi karier dan kehidupan pribadi seseorang*) |  |  |  |  |  |  |
| 28 | Know that it is inadmissible to transfer information about one's passwords, codes (in Internet and bank)  (*Mengetahui bahwa mentransfer informasi tentang kata sandi, kode seseorang (di Internet dan bank) adalah tidak dapat diterima secara moral*) |  |  |  |  |  |  |
| 29 | Are able to determine the degree of confidenality and security of the transfer of personal data when using the services via the Internet  (*Mampu menentukan tingkat kerahasiaan dan keamanan transfer data pribadi saat menggunakan layanan melalui Internet*) |  |  |  |  |  |  |
| 30 | Are able to downloaded and updated the soware  (*Mampu mengunduh/men-download dan memperbarui/meng-update software*) |  |  |  |  |  |  |
| 31 | Played online games  (*Mampu bermain game online*) |  |  |  |  |  |  |
| 32 | Know exactly how to buy and order goods and services in Internet shops  (*Mengetahui persis cara membeli dan memesan barang dan jasa di toko-toko Internet*) |  |  |  |  |  |  |
| 33 | Are able to look for work through the Internet  (*Mampu mencari pekerjaan melalui Internet*) |  |  |  |  |  |  |
| 34 | Are able to receive payment or transfer with electronic money  (*Mampu menerima pembayaran atau melakukan transfer dengan uang elektronik*) |  |  |  |  |  |  |
| 35 | Are able to managed a bank account through the Internet  (*Mampu mengelola rekening bank melalui Internet (Internet banking)*) |  |  |  |  |  |  |
| 36 | Know how e-government services work  (*Mengetahui bagaimana layanan e-government bekerja*) |  |  |  |  |  |  |
| 37 | Can change personal passwords on PCs and online services  (*Dapat mengubah kata sandi pribadi pada PC dan layanan online*) |  |  |  |  |  |  |
| 38 | Are able to delete the “history” of their actions on the Internet  (*Dapat menghapus “riwayat” aktivitas di Internet*) |  |  |  |  |  |  |
| 39 | Are able to change the settings of access to their information in social networks for different groups of users  (*Dapat mengubah pengaturan akses ke informasi di jejaring sosial Anda untuk berbagai kelompok pengguna yang ingin mengakses info tentang Anda*) |  |  |  |  |  |  |
| 40 | Can create mulple user accounts on the same PC or online service  (*Dapat membuat beberapa akun pengguna di PC atau layanan online yang sama*) |  |  |  |  |  |  |

INSTRUCTION:

Bring to mind a nostalgic event in your life. Specifically, try to think of a past event that makes you feel most nostalgic

(*Pikirkanlah peristiwa di masa lalu yang paling berkesan dan menimbulkan perasaan Nostalgia pada diri Anda.*)

Thinking about this event . . .

(*Saat mengingat pengalaman Nostalgia ini, …….*)

| **No** | **Statement** | **Strongly disagree** | **Disagree** | **Somewhat disagree** | **Somewhat agree** | **Agree** | **Strongly agree** |
| --- | --- | --- | --- | --- | --- | --- | --- |
| 1 | makes me feel happy  (*Saya merasa senang*) |  |  |  |  |  |  |
| 2 | puts me in a good mood  (*Suasana hati saya menjadi lebih baik*) |  |  |  |  |  |  |
| 3 | makes me feel active  (*Saya merasa bersemangat*) |  |  |  |  |  |  |
| 4 | makes me feel calm  (*Saya merasa tenang*) |  |  |  |  |  |  |
| 5 | makes me value myself more  (*Saya lebih menghargai diri sendiri*) |  |  |  |  |  |  |
| 6 | makes me feel like I have many positive qualities  (*Saya merasa seperti saya memiliki banyak kualitas positif*) |  |  |  |  |  |  |
| 7 | makes me feel good about myself  (*Saya merasa nyaman dengan diri saya sendiri*) |  |  |  |  |  |  |
| 8 | makes me like myself better  (*Saya merasa seperti diri saya yang lebih baik*) |  |  |  |  |  |  |
| 9 | makes me feel loved  (*Saya merasa dicintai*) |  |  |  |  |  |  |
| 10 | makes me feel connected to loved ones  (*Saya merasa terhubung dengan orang yang saya cintai*) |  |  |  |  |  |  |
| 11 | makes me feel protected  (*Saya merasa terlindungi*) |  |  |  |  |  |  |
| 12 | makes me feel I can trust others  (*Saya merasa bisa mempercayai orang lain*) |  |  |  |  |  |  |
| 13 | makes me feel that life is worth living  (*Saya merasa bahwa hidup ini layak dijalani*) |  |  |  |  |  |  |
| 14 | makes me feel life is meaningful  (*Saya merasa hidup itu bermakna*) |  |  |  |  |  |  |
| 15 | makes me feel life has a purpose  (*Saya merasa hidup ini memiliki tujuan*) |  |  |  |  |  |  |
| 16 | makes me feel there is a greater purpose to life  (*Saya merasa ada tujuan hidup yang lebih besar*) |  |  |  |  |  |  |
